# Accuracy Testing of Torque Limit Determination Algorithm Intended for Smart Bone Screwdrivers

**DOI:** 10.3390/s25133863

**Published:** 2025-06-21

**Authors:** Jack A. Wilkie, Alberto Battistel, Paul D. Docherty, Niklaus F. Friederich, Georg Rauter, Knut Möller

**Affiliations:** 1 Institute of Technical Medicine, Hochschule Furtwangen University, Jakob-Kienzle-Strasse 17, 78054 Schwenningen, Germany; alberto.battistel@hs-furtwangen.de (A.B.); moe@hs-furtwangen.de (K.M.); 2 Department of Biomedical Engineering, University of Basel, Hegenheimermattweg 167B/C, 4123 Allschwil, Switzerland; niklaus-f.friederich@unibas.ch (N.F.F.); georg.rauter@unibas.ch (G.R.); 3 Center for Bioengineering, Department of Mechanical Engineering, University of Canterbury, 69 Creyke Road, Christchurch 8041, New Zealand; paul.docherty@canterbury.ac.nz; 4 Department of Microsystems Engineering, University of Freiburg, Georges-Koehler-Allee 103, 79108 Freiburg im Breisgau, Germany

**Keywords:** bone screw, parameter identification, smart screwdriver, torque control

## Abstract

Bone screws are used in orthopaedic surgery for fracture fixation. Correctly torquing the screws is important for fixation quality. Over-tightening may strip the threads, while under-tightening may result in loosening over time. This paper focuses on testing an approach where strength is estimated using screw insertion data from torque and rotation sensors, and stripping torque is predicted based on this strength. A common type of bone screw was inserted until stripping 10 times each into 8 types of polyurethane surrogate for bone. The torque–rotation data from the insertion was used to identify the material strength and estimate the stripping torque and compared with the experimental maximum torque. A good relationship was found between the estimated/predicted and true stripping torques (r = 0.926, 95% confidence interval (C.I.) [0.886, 0.952]), with a mean error of 18%. Additionally, the intermediate identified strength values were found to be highly correlated with the data-sheet values for the materials (r = 0.977, 95% C.I. [0.964, 0.985]). These outcomes demonstrate the viability and significance of this concept in general, although more development and testing is required for broad clinical applicability; such tests would be extended for more types of bone screws and use a large set of human bone samples to better reflect the natural variability.

## 1. Introduction

Bone screws are used in a variety of orthopaedic procedures, primarily for fracture fixation. There also remains some use in implant fixation [1] despite many implant procedures using bone cement or other screw-less methods [2]. In fracture fixation, screws may be used alone [3], with plates or other more complex devices [4,5].

In general, bone screw fixations should be strong and long-lasting. These aspects may be affected by incorrect torquing. Over-tightening may result in thread stripping, which compromises the strength of the fixation [6], and the excessive mechanical pressure may compromise osteoblast viability [7], leading to loosening if the osteoclast-absorbed bone is not replaced. Meanwhile, under-tightening may lead to the screw loosening when subjected to cyclic loading or vibration [8]. Insecure implants from over- and under-tightening may also result in damage to the surrounding tissues [9] or additional disability. In extreme cases, revision surgery is required.

The use of locking screws and plates in modern surgery somewhat reduces the importance of careful torque control. However, locking screws are not always suitable, particularly since they cannot be used to axially compress bone fragments, they limit the angles of screw insertion, and they may be difficult to remove under some conditions [10].

Current surgical procedures generally involve the surgeon using their professional experience and tactile feedback to regulate screw torque. While this is successful in many cases, it is a subjective process, and human factors like stress and fatigue, as well as varying levels of experience and dexterity, may result in incorrect torquing [11]. Additionally, future technologies like semi- or fully-automated robotic surgery may exclude the surgeon from the torque-regulation process, so a quantitative system would be required. Furthermore, there are broad trends in the healthcare field towards using information from a multitude of sensors in combination with computational and AI models to extract new insights and improve patient outcomes [12].

Previous work proposed a physical model-based system of torque regulation [13]. This system consisted of two major steps: first, a model of the screw insertion is used with parameter identification to determine the bone strength based on torque-rotation signals from the screw insertion [14], which can be captured on a handheld device using strain gauges and a gyroscope [15]; then, the strength is used with a model of thread stripping to predict the stripping torque [16]. This prediction can be used with an appropriate limit (e.g., 80% of maximum, which can be optimized to statistically avoid both under- and over-tightening) and can be implemented with a visual torque-limit indicator (e.g., ring of coloured LEDs) or an electro-mechanical torque limiter (e.g., electromagnetic clutch). This system has the potential to increase the speed and safety of surgery while potentially requiring less training for new surgeons.

Other techniques have been proposed in the literature. Reynolds et al. [17] demonstrated a system that models the stripping torque as a multiple of the steady-state plateau torque characteristic of lag-screws (partially threaded with smooth shank). Thomas et al. [18] proposed a system that detects the tightening of screws via a spike in the time derivative of torque, which prevents stripping but does not necessarily guarantee a minimal torque. Wright et al. [19] showed a relationship between acoustic emissions from a screw during insertion and stripping torque and later demonstrated this can be used to reduce the occurrences of screw stripping [20]. While these methods are empirical models, the approach investigated here is novel because it is based on structural mechanics models derived from physical principles, and the development process may provide a better understanding of the bone-screw dynamics; additionally, with this method, the model is posed in terms of real physical parameters instead of lumped/empirical parameters/coefficients. To further motivate this method, Fletcher et al. [21] showed that even a simple digital torque-limit-indicating screwdriver, in general, is useful for reducing stripping, even when only a fixed limit was used based on a priori information, as opposed to individualized torque-limit determination of this current paper.

Our previous work focused on testing two separate stages of the torque prediction in isolation from each other [16,22] and only tested them as parts. In this paper, we will examine the combined system using a bone screw inserted into pre-drilled rigid polyurethane bone surrogates [23]. The screws will be inserted until failure to collect data for the stripping-torque estimation, and to record the true failure torque for comparison. The relationship between the estimated and true torques will be examined over eight types of polyurethane foam to test the viability of this technique.

## 2. Materials and Methods

### 2.1. Overview

This subsection is a brief overview/background to facilitate understanding the methods.

The overarching aim is to predict the stripping torque of the screw based on data during screw insertion. This is done based on torque and rotation readings during insertion. Generally, the bone strength is unknown, so this is determined by fitting a model, which normally predicts torque from a given rotation strength as a parameter (Equation (1)); but, by fitting the model to the measured torque and rotation, the strength parameter is determined. This strength is then used to predict the stripping torque with a second model (Equation (10)).

Here, the system is tested by systematically collecting torque–rotation data from screw insertions in a variety of materials. The data from before the tightening is used, as stated above, to predict the stripping torque. This is then compared to the actual measured stripping torque after tightening, and the relationship between actual and predicted stripping is analyzed.

### 2.2. Materials and Data Collection

Experimental data was collected by inserting a bone screw into rigid polyurethane foam using a purpose-built test rig.

**Figure 1:**
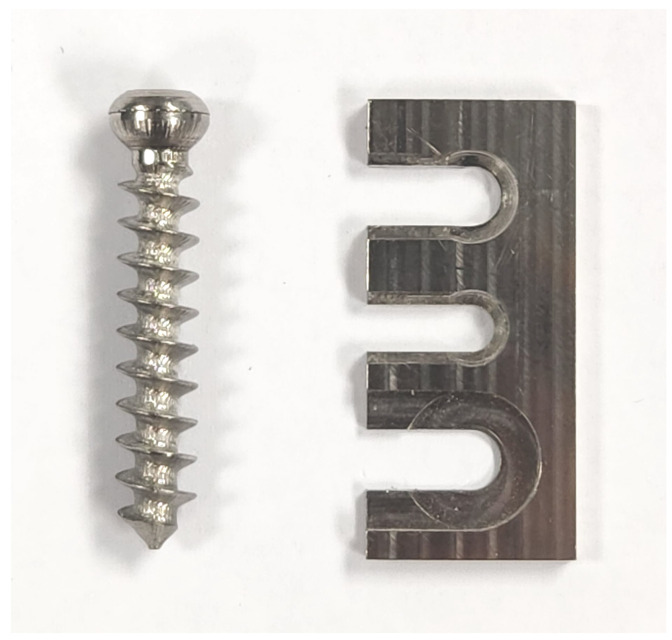
Screw and plate used for testing. The top hole of the plate matched the spherical underside of the screw.

The screw type used was an ISO 5835:1991 [24] HB 6.5 cancellous bone screw with a spherical surface under the head, 30 mm thread length, and machined from grade 5 titanium stock. The screw was tightened against a slotted grade 5 titanium plate with a matching spherical counter-sink. These are shown in Figure 1.

Eight densities of polyurethane foam were used in the testing; these are listed in Table 1. The test-blocks were prepared by cutting the stock into approximately 30 mm × 50 mm × 300 mm blocks. Then, through-holes were drilled on the 30 mm × 300 mm face, 10 mm from both long edges, using a 3 mm drill bit, with 10 mm spacing. Screws were inserted into the side of the drill entry to avoid any rear-side damage from the drill break-through.

**Figure 2:**
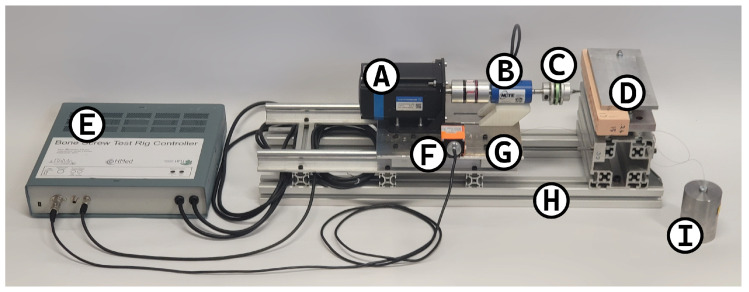
Test rig with labelled parts. A: Closed loop stepper motor. B: Rotational torque sensor. C: Screwdriver bit holder. D: Sample holder/clamp. E: Control box. F: Draw-wire encoder. G: Sliding carriage. H: Base. I: Weight for axial force.

The insertion was performed using a bespoke test rig shown in Figure 2. This uses a closed-loop position-controlled stepper motor (34HS46-6004D-E100 with CL86T driver, OMC Corporation Limited, Nanjing, China) to drive the screw into the material in a repeatable manner. A 2 kg weight attached to the sliding carriage through a pulley is used to apply axial force to the screw. A rotational torque sensor (NCTE-2300-5-1-AU-0-0, NCTE AG) is used to measure the torque applied to the screw and the rotation of the screw. The torque measurement has a ±5 Nm range, with 0.5% accuracy, while the rotation encoder has 360 CPR, giving 0.25 degree resolution with quadrature signalling. A draw-wire encoder (A40/D5.2501.2421.1000, Fritz Kübler GmbH, Villingen-Schwenningen, Germany) was used to measure the linear position of the screw, it has a 0.025 mm resolution with quadrature signalling. A control box contains the motor driver, 350W AC-DC power supply, and a microcontroller for real-time control, signal processing, and communications with a connected computer.

The modelling and test-rig measurements center around the torque and rotation signal, as these are able to be recorded by a handheld screwdriver in a clinical setting [15].

The screw was inserted into each of the 8 materials 10 times (to gain insight into the distributions of the real and estimated stripping torques), each time into a new hole (80 insertions total). Before each insertion, the position/rotation of the screw was zeroed at the point where the tip first enters the hole; this was done consistently by zeroing with an offset while placing the screw against a plate with known thickness that covered the hole. Then the screw was left to settle in the hole under the axial force of the 2 kg counterweight. The data recording was then started and the insertion was triggered. The test rig inserted the screw at 30 RPM until the threads were fully stripped. Then, the screw was automatically unscrewed for use in the next test before the recording was stopped. For the insertions into the higher density foam blocks, additional axial force was required to initiate screw engagement. This was provided by manually pressing against the back of the motor for the first 1 or 2 revolutions.

### 2.3. Data Processing

The data was pre-processed to crop it to the correct time period and segmented as described in more detail by Wilkie et al. [34]. The start of the experiment was determined by the screw beginning to rotate and passing 1 degree from it’s start position. The end was defined as when the insertion rotation stopped (when the rotation first reached within 1 degree of its maximum value). Additionally, the point of stripping was defined as when the torque reached its maximum value. To determine the point where the screw began to tighten, the torque data was smoothed with a 1000 sample (1 s) moving average filter, numerically differentiated, and the tightening point was defined as when this derivative first exceeded 6 times the 90th percentile of this derivative. For the parameter identification/stripping torque estimation below, only the insertion segments were used (i.e., before tightening began). The full data was used to find the actual/experimental maximum torque.

The linear position was a more reliable indicator of screw depth than rotation, due to the slippage of the screw during the initial thread engagement with the hole. However, as the model below uses rotation, the linear position data was converted into the equivalent rotation based on the pitch of the screw and used for the rotation data.

For determining the estimated stripping torque, only the segments of data before the tightening was used. First, the torque–rotation data from each screw insertion (not tightening/stripping) was used to fit the model of the screw insertion with linear least squares. Generally, the model is based on the principle that the screw surface in contact with the hole material will experience a normal compressive stress due to the forcibly-deformed hole material (bone/PU-foam), roughly equal to the yield stress of the hole material (as the yield stress was reached to deform the material, and no de-loading occurs after). This normal force results in a tangential/shear friction stress along the screw surface dependent on the normal stress and friction coefficient. Carefully integrating this friction/shear stress times the radius from the screw center-line over the surface of the screw then gives the total friction torque. There is also similar cutting torque based on the radial stress applied by the tapered thread at the end of the screw as it deforms the hole to form threads; although this torque is at least an order of magnitude smaller in our case.

**Figure 3:**
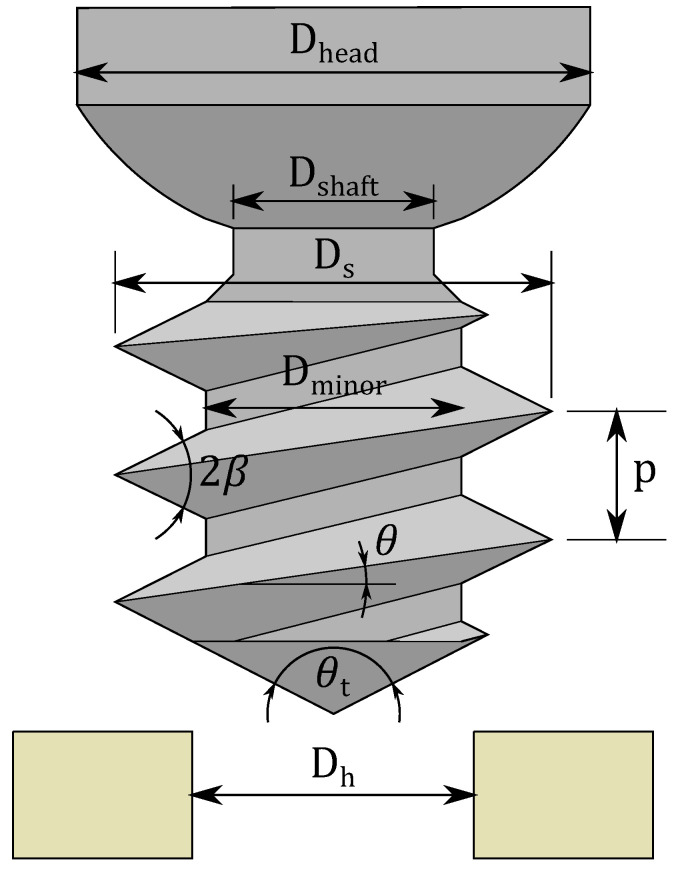
Geometric representation of the parameters used in the modelling.

The mathematical screw insertion model is shown in Equations (1)–(9). Torque ($\tau_{\mathrm{total}}$) was modeled as a function of rotation (ϕ), with known parameters based on the screw and hole geometry (shown visually in Figure 3 and listed in Table 2) and the unknown parameter of material strength ($\sigma_{\mathrm{ucs}}$). The value of $\sigma_{\mathrm{ucs}}$ is determined through the fitting process. Parameters relating to cutting torque are combined into $G_{1}$, while those relating to friction torque are combined into $G_{2}$. $r_{s}$ and $r_{f}$ are the effective radii that cutting and friction forces act through, respectively. $A_{c}$ is the thread cross-sectional area that intersects the hole wall. $K_{f0}$ is a geometric parameter relating screw geometry to friction forces. $D_{h}$ is the hole diameter. $D_{s}$ is the screw major diameter. θ is the thread pitch angle of the screw, also based on the pitch, *p*. β is the thread profile half-angle. $\mu_{T}$ is the friction coefficient between the screw threads and the hole. This model was introduced in Wilkie et al. [35], and was based on Seneviratne et al. [36].

(1) $$\tau_{\mathrm{total}}=\tau_{\mathrm{friction}}+\tau_{\mathrm{cutting}}$$

(2) $$\tau_{\mathrm{total}}=\sigma_{\mathrm{ucs}}G_{1}+\mu_{T}\sigma_{\mathrm{ucs}}\left(\phi -\frac{\alpha }{2}\right)G_{2}$$

(3) $$G_{1}=r_{s}A_{c}\cos\theta$$

(4) $$G_{2}=2r_{f}K_{f0}\cos\theta$$

(5) $$r_{f}=\left(D_{h}+D_{s}\right)/4$$

(6) $$r_{s}=\left(2D_{h}+2D_{s}\right)/6$$

(7) $$K_{f0}=\frac{1}{2}\left(D_{s}-D_{h}\right)\times \sqrt{\left(1+tan^{2}\beta \right)\left[{\left(\frac{D_{s}+D_{h}}{4}\right)}^{2}+{\left(\frac{p}{2\pi }\right)}^{2}\right]}$$

(8) $$A_{c}=\tan\beta {\left(\frac{D_{s}-D_{h}}{2}\right)}^{2}$$

(9) $$\theta =\tan^{-1}\frac{p}{\pi D_{s}}$$

After the material strength is identified with the above model by a least-squares fit with the pre-tightening experimental data, each strength value is converted into an estimated stripping torque using the model from Wilkie et al. [16], which is summarized in Equations (10)–(14). This model has $D_{\mathrm{head}}$ as the diameter of the screw head contact surface; $D_{\mathrm{shaft}}$ as the diameter of the screw shank at the head of the screw; $\mu_{H}$ as the friction coefficient between the screw head and surface/plate; $\theta_{\tau }$ as the angle of the shear stresses on the cylindrical envelope of the screw; $A_{C}$ as the surface area of the cylindrical screw envelope, *L* as the threaded length of the screw; and $D_{\mathrm{minor}}$ as the minor diameter of the screw/thread. The equation for $\tau_{\mathrm{fail}}$ does not include a stress concentration factor ($k_{t}$) as written, but this can be applied by dividing the resultant failure torque by the concentration factor. The stress concentration factor incorporates the effects of sharper or blunter thread tips on the stripping of the threads; however, it would be expected to be more relevant to brittle materials where material failure leads to propagating cracks, more so than ductile materials where failure leads to the material more slowly yielding.

(10) $$\tau_{\mathrm{fail}}=\frac{\sigma_{\mathrm{ucs}}}{\sqrt{3}}A_{C}\times \left(\frac{1}{3}\frac{D_{\mathrm{head}}^{3}-D_{\mathrm{shaft}}^{3}}{D_{\mathrm{head}}^{2}-D_{\mathrm{shaft}}^{2}}\mu_{H}\sin\left(\theta_{\tau }\right)-\frac{D_{s}}{2}\cos\left(\theta_{\tau }\right)\right)$$

(11) $$\theta_{\tau }=\mathrm{atan}2\left(p,\pi D_{s}\right)+\mathrm{atan}2\left(1,-\mu_{T}\right)$$

(12) $$A_{C}=\pi D_{s}L$$

(13) $$k_{t}=1+\left[1+1.5\tanh\left(0.3\ln\gamma +0.7\right)\right]\sqrt{\gamma }$$

(14) $$\gamma =(D_{\mathrm{shaft}}-D_{\mathrm{minor}})/p$$

### 2.4. Data Analysis

The resulting estimated stripping torques from above were compared with the true stripping torques. The true stripping torques were found by filtering the torque signal with a 30-sample median filter (0.03 s), then selecting the maximum torque value from each test.

The combined dataset (all materials estimated vs. true stripping values) was analysed by linear regression. The relationship between identified and the data-sheet strength values in Figure 1 was also similarly analysed.

### 2.5. Flexural Testing

**Figure 4:**
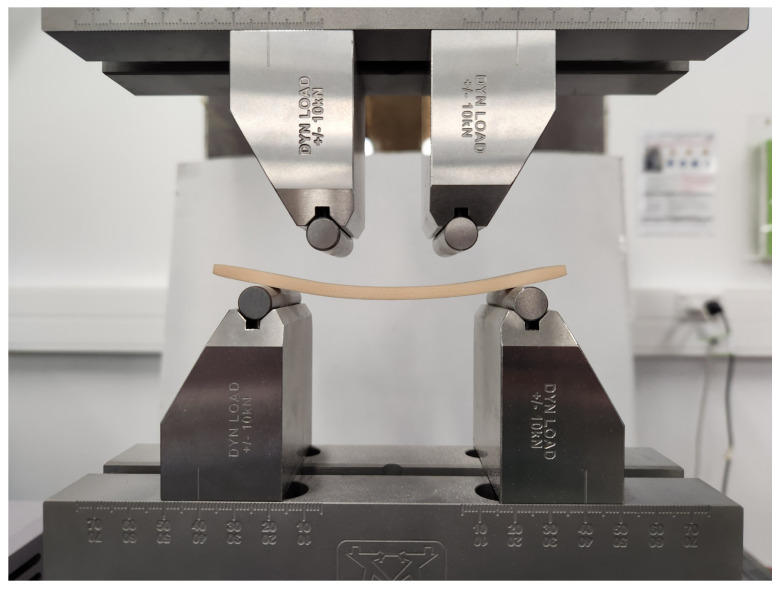
Bend testing setup used to quantify ductility of PU foam samples. Currently showing remaining plastic deformation in a sample after deloading.

Six of the materials (M330, M450, M600, 15 PCF, 20 PCF, 30 PCF) were tested for ductility with 4-point bend testing. This is reported in detail by Wilkie et al. [39]. The method was based on the method in ASTM D6272 - 17. The samples were 14.6±0.1 mm wide and 5.1±0.2 mm thick, with a support span of 80.0±0.2 mm and a load span of 40.0±0.2 mm with a cross-head rate of 10 mm/min. The spans and each samples’ dimensions were measured to ±0.05 mm with digital calipers. For ductility measurement, the materials were progressively deformed and relaxed with increasing maximum strain until failure. The ductility was calculated as the percentage of plastic deformation (remaining after unloading) compared to the total deformation at failure. The testing was performed on an MTS Criterion™ Model 43 with a 500 N load cell with ISO 376 accuracy class 1 from 5–500 N (0.5% uncertainty) and an Instron 10 kN dynamic four-point bend test fixture (P/N 2810-500/505), shown in Figure 4.

## 3. Results

**Figure 5:**
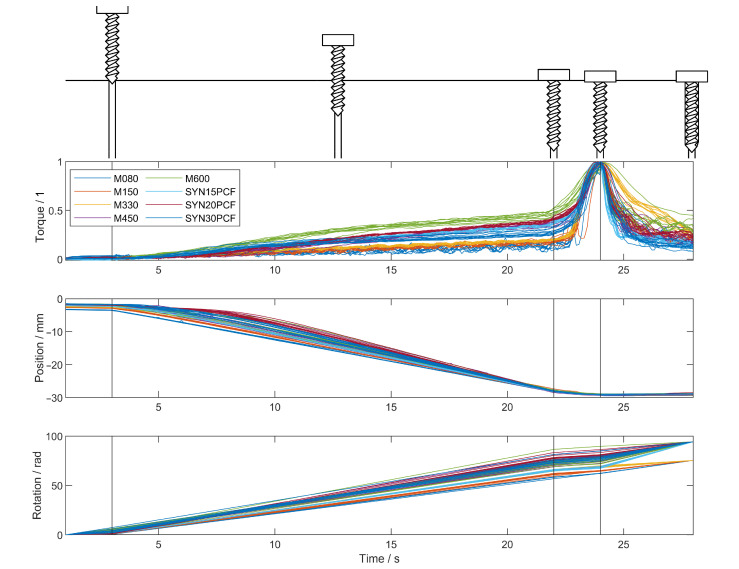
Cropped data from experiment. Different segments between black lines were linearly time-rescaled to fit in the same area despite the true times being different. All torques were normalised by the maximum/stripping torque value. The segments can be labelled from left to right as: engagement (N.B. narrow), insertion, tightening, stripping.

To verify the correct segmentation of data, all cropped datasets were plotted on the same axes, as shown in Figure 5. To make the data more comparable in the illustration, the torque was normalised based on the empirical stripping torque. All datasets show correct segmentation when the screw begins to tighten, with minimal inclusion of the insertion segment in the tightening segment. Two trials (with M080 and M150 in Figure 5 Torque) deviate slightly from the main trend in the tightening segment, with an extra slight peak and trough; this was from the screw not seating properly into the plate used, then suddenly slipping into the correct position causing a momentary loss of compression and plate-head friction torque. As the harder materials were slower to engage the screw threads, a larger rotation endpoint was used to ensure they indeed fully inserted and stripped, as seen at the end of the rotation plot in Figure 5. It is also clear that the ratio of the final torque before tightening begins to the stripping torque is not constant across different materials, varying from about 0.2 to 0.5.

**Figure 6:**
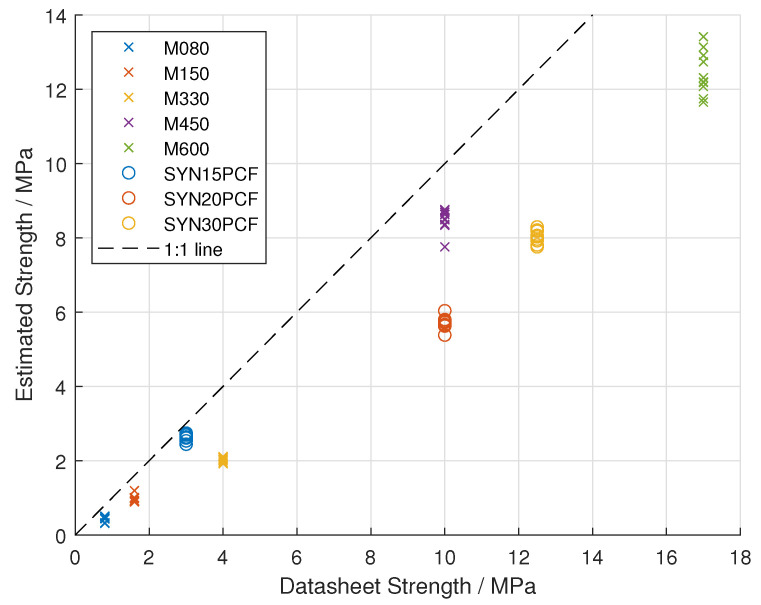
Results of strength prediction over 8 different types of polyurethane foam from 2 manufacturers compared to the data-sheet strength values. The datasets from the different densities of polyurethane foam are differentiated by colour.

The intermediate strength values (used to calculate the stripping torque) are also plotted in Figure 6. These are compared to the data-sheet values. The correlation with the data-sheet values is r = 0.977 (95% C.I. [0.964, 0.985]).

**Figure 7:**
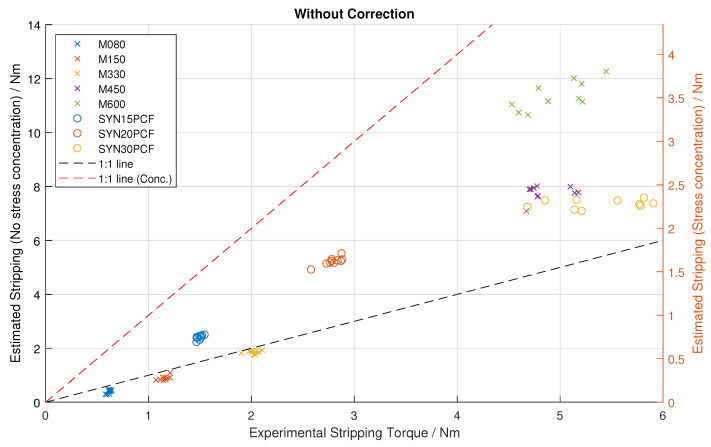
Results of striping torque prediction over 8 different types of polyurethane foam from 2 manufacturers. The left *y*-axis is without the stress concentration factor correction, and the right *y*-axis is with it. The datasets from the different densities of polyurethane foam are differentiated by color. 1:1 lines are plotted for both *y*-axis scales.

The results of the combined stripping-torque prediction are shown in Figure 7. The prediction can be performed by excluding the stress concentration factor, which is more relevant to ductile failure, or including the factor, which better represents brittle failure. As this factor only scales the prediction, both are shown on the same plot with two y axes; this also leads to two 1:1 lines (one for each scale/prediction variant). The r-coefficient for the true vs. estimated stripping torque data (In Figure 7) is 0.926 (95% confidence interval: [0.886, 0.952]). The correlation for the strengths vs. the data-sheet is statistically significantly stronger than the correlation for the true stripping torques vs. the estimates with a Fisher Z-test score of *p* = 0.0002.

**Figure 8:**
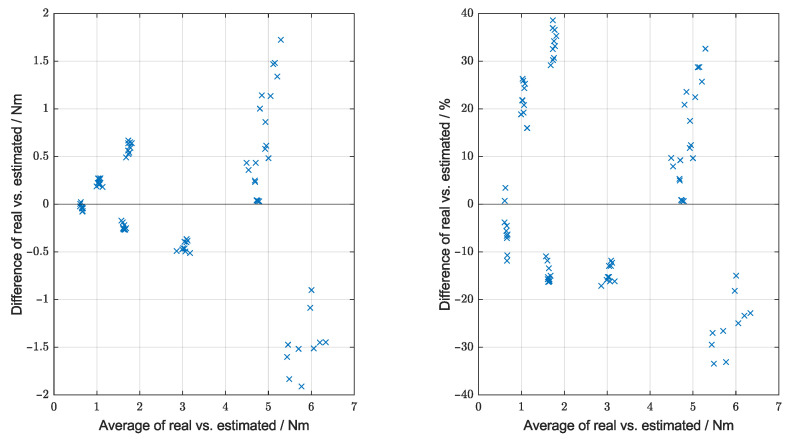
Bland–Altman plots of the stripping torque estimations. Left showing the absolute error and right showing the relative error.

The results are also presented in the form of a Bland–Altman plot in Figure 8 to show the error distribution. As the model would be calibrated in practice, the results were corrected using a linear calibration before being plotted. As the results show the error is proportional to the magnitude of the data, a proportional plot is also given. The average relative error is 18.0%.

The percentage ductilities from the 4-point bend testing are listed in Table 3.

## 4. Discussion

Figure 7 shows a clear relationship between the estimated (model-based prediction) and true (experimental) stripping torques. This is also shown with the high correlation coefficient of 0.926. Additionally, there was a high correlation between the identified and reference strengths of 0.977. The correlation between the identified vs. data-sheet strength is also higher than the correlation between estimated and experimental stripping torque (Fisher Z-test p = 0.0002), implying that some inaccuracy is introduced in the first step (strength identification) of the system and then further inaccuracy is introduced in the second step (stripping torque prediction).

Figure 7 shows that the proposed method can predict the stripping torque of bone screws based on the intra-insertional torque–rotation signals with a degree of precision required for clinical use. This method therefore shows promise for use in a clinical context to regulate bone screw tightening torque in an automatic and objective manner. While a special test setup was used for consistency in this experiment, these signals can be recorded clinically, as shown already in a prototype system [15]. If successfully developed, this approach has the potential to improve patient outcomes or allow advances like robotic screw insertion. Additionally, the high correlation of the identified strength values with the known values gives confidence in the usefulness of a mechanical model compared with an empirical one; as the strength value alone may be useful in a clinical context even if the torque regulation concept is not used. For example, the strength value could be used to estimate if the number of screws used is sufficient for a then-known bone strength and expected loading, and the clinician could decide intra-operatively to enhance the fixation with additional screws if necessary.

Figure 8 quantifies the accuracy of the model over the tested range. The error is clearly proportional to the magnitude of the data; however, that is not an issue because the margin for error, being a percentage of the total torque, is also higher for screws in hard materials with higher stripping torques. The more important percentage error plotted in Figure 8 shows a range of roughly −35% to +40%, with an average absolute error of 18.0%. Ideally, the model should be further developed to improve this performance (some factors and discussed below), as using a standard “70% of maximum” guidance [40] will result in stripping at times (if the error is over 30%); although comparison with the status-quo (manual human tightening) is also important, as even a non-perfect estimate may potentially be better than a human. It may also be directly usable if the limit is set at 60% of the maximum instead; however, this also increases the chances of the less serious under-tightening (less serious compared to irreversible over-tightening, as it can be resolved by further tightening if detected).

The model presented has the option to incorporate a stress concentration factor in calculating the stripping torque. This represents how the sharper screw edges locally increase the stresses induced in the bone, which leads to yielding lower loads than would be predicted without using the stress concentration factor. This yielding will behave differently depending on the material; in ductile materials, the yielding will first lead to plastic deformation and load redistribution, allowing higher loads than the stress concentration factor implies before ultimate failure, while in brittle materials, the yielding may lead to a more immediate failure due to prompt fracture formation and propagation. This means that ductile failure is more dependent on the average stress exceeding the limit, while brittle failure is more dependent on the peak concentrated stress. The separation of this factor in the model allows this effect to be fine-tuned in the future. As Figure 7 shows, all of the failures occurred near or between the 1:1 lines; this means that ignoring the stress concentration ranged from accurate to optimistic, and the full inclusion was pessimistic, with most data points lying between the two extremes. If the stress concentration variation is assumed to be the only effect, this suggests that most materials lay somewhere between the two extremes. From the ductility testing, it was seen that the materials closer to the 1:1 line of the stress concentration scale were generally the more brittle ones, while the ones close to the 1:1 of the no stress concentration scale were the more ductile ones, as expected. While this could be investigated further, there is little utility in over-analysing the effect in PU foam when the eventual application in bone may be markedly different. However, a possible speculative trend may be that denser, stronger, and more mineralised bone tissue may be more brittle, so a correction curve could be used to apply a proportion of the stress concentration factor in the torque prediction step based on the identified strength.

The testing performed here also shows that the method works over a range of material densities and manufacturers. This suggests that it is adaptable and provides increased confidence that it will function well in bone, which is even more variable.

While the testing shows a good relationship, it is clearly not perfect. There is some spread for the groupings of the individual materials, and the groups are not centred in a perfectly straight line. For the groupings, manufacturing variation (both in the bulk material properties and variations in the hole-drilling process) will cause some variation in the true stripping torques; this is also expected to cause correlated variation in the estimated torques, although the estimates will have additional spread due to errors incurred from measurement and estimation of the stripping torque. As previously stated, the variation in ductility appears to affect the variation in group means away from a linear relationship; however, other un-modelled aspects may also contribute (e.g., elasticity, varying material composition, micro-structure, etc.). As this paper only covers the general demonstration of this method as an effective way of predicting torque, a more thorough investigation of these effects and potential correction methods is recommended for future work in the field.

The greatest limitation of this study is the exclusive use of PU foam as a substitute for bone. Real bone will certainly have different properties and may require model adjustments (particularly the friction coefficient and stress concentration effect magnitude). However, there are a number of advantages to testing with PU foam. The consistency of the material allows inspection of the effects of measurment noise on the estimation. In particular, Figure 7 shows that the estimations from each material are tightly clustered. This demonstrates the consistency of this method. Such an analysis is impossible with real bone due to the uncontrollable magnitudes of variation in bone strength. The data-sheet availability also provides a useful reference for comparison of the intermediate strength values generated by the system. While bone strength can be measured, strength measurement is generally a destructive process and it would not be possible to perform strength testing and screw insertion at the same point on the same sample. Using different samples or points on the same sample would be insufficient due to the inter- and intra-sample variability of bone.

Additionally, the model assumes a standard bilinear-elastic material model with isotropic and homogeneous properties. Specifically, the homogeneous assumption is violated by certain types of bone, where the density of cancellous bone can vary along the length of the bone–screw interface (1), there can be a layer of denser cortical bone at the surface (2), or in the shafts of bones (for bi-cortical fixation) there can be layers of dense cortical bone on each side of the bone with essentially non-fixating bone marrow in the middle (3). As this method is more intended for cancellous bone, the bi-cortical case (3) is less critical as a limitation of this method; however, the other cases (1/2) remain important to discuss. As it stands, the model (used naively as is) would be expected to give an average material strength of the bone along the bone–screw interface, as each bone section contributes to the overall torque proportionally to its strength, and these effectively weighted average torque contributions control the total averaged strength estimate. Hence, the varying density should be portrayed as a strength estimate that shows the weighted average of bone strengths along the screw fixation, and it should give a reasonable stripping torque prediction. This, of course, still remains to be verified with varying-density test samples. One potential pitfall of this is in the effects of ductility for stripping torque prediction: for simple varying-density cancellous bone (1) the ductility may be expected to be similar along the length, causing fewer complications, but in the case of a dense/cortical surface layer (2), the ductility difference may be substantial. To capture the two layers’ properties independently for more careful consideration, the model could be updated with a minimally parameterized strength as a function of depth (minimal parameters are important to maintain practical identifiability), for example, with two strength values and a transition depth. This would also necessitate a more complex non-linear identification algorithm like gradient descent or some annealing algorithm instead of the simple linear least squares method used in this paper. These strengths can then be used independently to calculate their contributions to the stripping torque, while considering the potentially differing effects of ductility for each segment individually.

The values for friction used in the parameter identification here were based on the existing literature. Friction coefficient values are not trivial to measure as they may depend on factors such as pressure and surface speed, surface roughness, and moisture levels. To apply these results to real bone, at the very least, these coefficients should be re-examined. Further studies should also examine the variability of friction values in practice. Since the friction coefficient almost directly correlates torque with material strength in the insertion phase, variation in the friction coefficient will almost inversely proportionally affect the identified strength and stripping torque; this could be especially affected by the surface roughness of the screw and bone (although the screw surface is easier to control) and the wetness of the interface (bodily fluids). Future work may also uncover ways to compensate for the variability or control the influencing factors (e.g., flooding the screw site with a uniform known fluid like standard saline).

Overall, thorough testing on real ex vivo bone is recommended in future studies. Despite the aforementioned advantages of PU foam testing, real bone would allow testing with biologically accurate friction and ductility and would provide more confidence in the system’s performance in a clinical context.

In this study, a constant axial force from a 2 kg weight was used to help the screw threads engage the hole. This was kept constant over all tests in order to help control as many variables as possible, although an additional initial push was required for the densest material tested to start screwing into the sample (described in methods). However, in the case of softer materials, a lower force would be ideal to prevent crushing the material so much during the initial engagement of the threads, and for the harder materials, a higher force is ideal to prevent the screw from initially just "drilling" out material; both cases can weaken the first turns of the threads formed in the sample. The screw used in this study has about 10 turns of thread, so this effect will only have a small effect on our results, but it is an important factor to consider in the implementation of this system. It is especially important in real bone, as the outermost layers are usually harder/stronger, so obtaining the correct initial axial force will optimize the fixation in this strong outer layer. This has been previously investigated in Wilkie et al. [41], where a model was developed and qualitatively tested to provide optimal axial forces based on known parameters and known material strengths; this could also potentially be incorporated into a larger system alongside the model of the current study to provide feedback on axial force as well as torque. However, there are still a number of barriers to overcome, such as the fact that the axial force recommendation is required at the start of the insertion, and the model described in the current study can only provide a strength estimate (which could be used to calculate optimal axial force) after the screw has advanced significantly into the material; but there may still be utility in using this estimate for subsequent insertions at the same or similar (e.g., adjacent or contralateral) anatomical sites, which would be expected to have similar properties.

Only a single screw size was used in this testing. Including additional sizes and shapes of screws would help to further validate this approach and would give more confidence when applying it to a previously-untested screw–material combination. As resources are always limited, it would be advisable to test the more extreme cases of size and shape parameters to avoid extrapolating results, and a representative sample of more average combinations to validate the interpolation of parameters. One example could be screws intended for cortical fixation; these generally have much shallower threads which would likely lead to lower and less consistent friction torque (as the same random variation in hole size would affect a larger proportion of the thread surface), and accordingly lead to more uncertainty in strength determination. Additionally, this is primarily intended for self-taping screws in cancellous bone, as cortical bone may not exert the same degree of residual stress on the screw thread, especially if a hole is tapped before the screw is inserted. The model used in this paper also only applies to uniform trapezoidally profiled screws; however, more complex and varying thread profiles could be implemented using a slightly more complex model formulation such as that derived in Wilkie et al. [14].

This work follows the principle of Human–Robot shared control, as outlined in Zhang et al. [42]. While the smart screwdriver being targeted as the output of this research is not specifically a robotic product, it combines some of the best aspects of pure human surgical practice (the most direct/intuitive control of the instrument, and high dexterity/adaptability of human motion), with some of the advantages of robotic surgical practice (quantitative control/limits, high accuracy measurements). Even though this research is not explicitly targeting robotic applications, the outcomes are easily transferred to the field of robotics (once further developed to improve accuracy as much as possible) as a torque controller/limiter on a robotic screwdriver end-effector instead of an inline torque indicator in a smart screwdriver. The dexterity of robotic arms and optimal/ergonomic/intuitive manipulation and surgical planning/registration is also still an open research field that would help the application of this method in robotic surgical practice.

## 5. Conclusions

A system to estimate the stripping torque of a screw based on torque–rotation signals from the insertion was tested. The system used a novel model-based method to estimate the strength and, in turn, stripping torque. The system’s predictions were shown to be highly correlated (r = 0.926) with the experimental stripping torques, with a mean error of 18%. A partial system was also shown to be effective at measuring the strength of bone as an additional utility.

While many steps are required before a clinically viable device is developed, this concept/system seems to be reliable and has the potential to improve patient outcomes in traditional surgery and allow adaptive torque–limitation in robot-driven screws. Future developments can focus on fine-tuning or calibrating the model, especially in real bone. Such testing on real bone is required to validate that the system works effectively in that case.

## Figures and Tables

**Table 1:** Material properties of the rigid polyurethane foam used. Real bone values given for comparison.

Material Name	ρ ($g\cdot {\mathrm{cm}}^{-3}$)	$\sigma_{\mathrm{ucs}}$ (MPa)	*E* (MPa)
${\text{SikaBlock}}^{®}$ M80 [25]	0.08	0.8	24
${\text{SikaBlock}}^{®}$ M150 [26]	0.16	1.6	65
${\text{SikaBlock}}^{®}$ M330 [27]	0.24	4	150
${\text{SikaBlock}}^{®}$ M450 [28]	0.45	10	430
${\text{SikaBlock}}^{®}$ M600 [29]	0.60	16–18	750
${\text{SYNBONE}}^{®}$ 15 PCF [30]	0.24	2–4	-
${\text{SYNBONE}}^{®}$ 20 PCF [30]	0.35	8–12	-
${\text{SYNBONE}}^{®}$ 30 PCF [30]	0.47	10–15	-
Cancellous Bone [31,32] (Femoral head)	0.61–0.88	0.15–21	345–1475
Cortical Bone [32,33] (Femoral shaft)	1.83–2.03	69	6900

**Table 2:** Parameters used in the parameter identification and stripping–torque estimation. Note that the screw thread friction coefficient used is based on applying the method to PU foam samples, and a different value should be found or otherwise determined to apply this method in bone.

Symbol	Value	Unit	Name
$D_{h}$	3.2	mm	Hole diameter
$D_{s}$	6.5	mm	Screw major diameter
$D_{\mathrm{shaft}}$	2.8	mm	Screw shank diameter
$D_{\mathrm{head}}$	7.0	mm	Head contact diameter
$D_{\mathrm{minor}}$	3.0	mm	Screw minor diameter
β	15	degrees	Screw thread half angle
*p*	2.75	mm	Screw thread pitch
$\mu_{T}$	0.2		Foam-screw friction co-eff. [37]
$\mu_{H}$	0.45		Plate-screw friction co-eff. [38]
$\theta_{t}$	60	degrees	Screw end taper

**Table 3:** Ductility values calculated for representative sample of materials.

Material	Ductility %
SikaBlock M330	26.7
SikaBlock M450	16.3
SikaBlock M600	19.2
SYNBONE 15 PCF	20.7
SYNBONE 20 PCF	14.4
SYNBONE 30 PCF	27.4

## Data Availability

Dataset available on request from the authors.
